# Healthy Community Design, Anti-displacement, and Equity Strategies in the USA: A Scoping Review

**DOI:** 10.1007/s11524-022-00698-4

**Published:** 2022-12-29

**Authors:** Natalicio Serrano, Lindsey Realmuto, Kaitlin A. Graff, Jana A. Hirsch, Lauri Andress, Mojgan Sami, Ken Rose, Akimi Smith, Katherine Irani, Jean McMahon, Heather M. Devlin

**Affiliations:** 1 Institute for Health Research and Policy, School of Public Health, University of Illinois at Chicago, Chicago, IL USA; 2 College of Urban Planning and Public Affairs, University of Illinois at Chicago, Chicago, IL USA; 3 Division of Nutrition, Physical Activity, and Obesity, National Center for Chronic Disease Prevention and Health Promotion, Centers for Disease Control and Prevention, Atlanta, GA USA; 4 McKing Consulting Corporation, Atlanta, GA USA; 5 Urban Health Collaborative and Department of Epidemiology and Biostatistics, Dornsife School of Public Health, Drexel University, Philadelphia, PA USA; 6 Department of Medical Education, Geisinger Commonwealth School of Medicine, Scranton, PA USA; 7 Department of Public Health, California State University Fullerton, Fullerton, CA USA; 8 Coordination Operations and Response Element, Administration for Strategic Preparedness and Response, U.S. Department of Health and Human Services, Washington D.C., USA

**Keywords:** Displacement, Neighborhood development, Active living, Health equity

## Abstract

Recent investments in built environment infrastructure to create healthy communities have highlighted the need for equity and environmental justice. Although the benefits of healthy community design (e.g., connecting transportation systems and land use changes) are well established, some reports suggest that these changes may increase property values. These increases can raise the risk of displacement for people with low incomes and/or who are from racial and ethnic minority groups, who would then miss out on benefits from changes in community design. This review scanned the literature for displacement mitigation and prevention measures, with the goal of providing a compilation of available strategies for a wide range of audiences including public health practitioners. A CDC librarian searched the Medline, EbscoHost, Scopus, and ProQuest Central databases, and we identified grey literature using Google and Google Scholar searches. The indexed literature search identified 6 articles, and the grey literature scan added 18 articles. From these 24 total articles, we identified 141 mitigation and prevention strategies for displacement and thematically characterized each by domain using an adapted existing typology. This work provides a well-categorized inventory for practitioners and sets the stage for future evaluation research on the implementation of strategies and practices to reduce displacement.

## Introduction

Neighborhood development has been described as a means to elicit social, economic, political, and environmental change in communities in response to dismal conditions and areas in decline [1]. Neighborhood development investments may be federally funded initiatives such as Community Development Block Grants, but can also be driven by community members or non-profit organizations [2]. Historically, these strategies have focused on improving social and economic outcomes [3], typically in the form of ensuring housing and providing social services. Recently, there has been a shift and focus on neighborhood development as a way to support built environment infrastructure for healthy living [2, 4, 5]. Specifically, there have been historic investments in infrastructure from the federal government, including the Bipartisan Infrastructure Law [6], Justice40 Initiative [7], and the American Rescue Plan [8]. These investments have been put in place to tackle issues of environmental justice, the COVID-19 pandemic, and the overall health and well-being of communities.

A specific example of how these infrastructure improvements may influence health includes the Community Preventive Services Task Force (CPSTF) recommendation for built environment approaches to increase physical activity that combine infrastructure modifications for transportation with changes in land use and community design, such as adding sidewalks and bicycle lanes, expanding public transit, improving parks and recreation facilities, and allowing residential density and mixed-use development that enables housing in proximity to destinations such as businesses and schools [9]. These recommendations have been translated by CDC’s Active People, Healthy Nation Initiative into a strategy called “activity-friendly routes to everyday destinations” and they may be especially beneficial to communities with people of lower incomes or who are from racial and ethnic minority groups, who some studies have shown may live in neighborhoods lacking built environment features that support physical activity (e.g., homes close to parks, schools and jobs; safe walking and biking routes; expanded public transit), while also facing disparities in car access [10–12]. Additionally, similar built environment strategies have been shown to mitigate urban heat island effects [13], and facilitate community resilience [14]. Communities with people of lower incomes or who are from racial and ethnic minority groups also have higher rates of physical inactivity and chronic diseases, such as cardiovascular disease [15, 16].

However, built environment modifications intended to improve health for communities with people of lower incomes or from racial and ethnic minority groups may result in differential negative impacts [17]. There are mixed findings on how infrastructure changes related to physical activity have impacted property values and displacement. The concept of residential displacement was proposed by Grier and Grier [18] and is described as occurring “when any household is forced to move from its residence by conditions which affect the dwelling or immediate surroundings, and which (1) are beyond the household’s reasonable ability to control or prevent; (2) occur despite the household’s having met all previously-imposed conditions of occupancy; and (3) make continued occupancy by that household impossible, hazardous or unaffordable.” Similarly, commercial displacement is the process by which low-value businesses become displaced with the introduction of higher value businesses or up-scale housing [19]. Physical displacement has relatively obvious, negative social and economic impacts, such as a loss of a preferred residence, social networks, housing stability, and educational opportunity. Studies on activity-friendly routes for bicycling showed a positive association with property values, but no association with displacement [20, 21]. Close proximity to light rail transit stations may drive displacement [22]. Additionally, increasing access to green space in urban areas has been linked to increased gentrification [23, 24]. The term “gentrification” is contested and has evolved across geospatial and historical contexts [25, 26]. The term was first used by the sociologist Ruth Glass in the 1960s, who described working class quarters of London being “invaded by the middle classes – upper and lower” [27]. While the original use focused on class, contemporary definitions of gentrification, particularly in the US context, incorporate both class and race and increasingly recognize structural economic inequities and social and political complexities. For example, a recent systematic review of gentrification and health outcomes research in the US-defined gentrification as “an interactive process in formerly declining, under-resourced, predominantly minority neighborhoods involving economic investment and increasing sources of capital infusion and in-migration of new residents, generally with a higher socio-economic status” [28]. Gentrification has also been studied as a process that may worsen existing health disparities [29–31]. For example, a recent systematic review on the health impacts of gentrification found that Black people and people with lower incomes living in gentrifying areas suffered negative effects including mental health disorders and poor self-rated health [32]. However, no long-term or robust assessments exist that document the impacts of investment in built environment infrastructure on gentrification or displacement.

Still, communities have valid concerns related to increased property values like increased rent or property taxes [20, 22–24]. To promote equitable distribution of costs and benefits in the context of built environment improvements to increase physical activity, efforts are needed to understand potential strategies to prevent displacement or mitigate its harms (hereafter “anti-displacement strategies”) when investing in physical activity infrastructure. Much of the previous literature on strategies to prevent or mitigate displacement has focused on affordable housing [33, 34]. However, affordable housing strategies may be insufficient to protect low-income residents in a context of increasing property values from which many cannot benefit [35]. Actions to prevent displacement may require multiple strategies utilizing a variety of tactics.

Therefore, the current review aims to (1) compile toolkits and resources to help practitioners learn about available anti-displacement strategies; (2) from these toolkits and resources characterize a broad range of anti-displacement strategies that have been proposed, implemented, and/or evaluated in the USA; and (3) discuss the implications of these strategies for centering equity in projects aiming to improve the built environment for healthy lifestyles.

## Methods

### Search Strategy

The present review consisted of a broad search for indexed literature, as well as a complementary grey literature scan. We consulted 10 experts in the field for background and context to inform our search strategy. To be included in this review, articles must have been published in the past 20 years (i.e., since 2002). This timeframe reflects the more recent emergence of literature in this field. The search strategy included key terms for “gentrification” or “displacement” or “urban renewal” and “mitigation” or “prevention”. Indexed publications were identified in November 2021 by a CDC librarian from searches of the Medline, EbscoHost, Scopus, and ProQuest Central databases. A complementary grey literature scan via Google and Google Scholar was commenced in May 2018 and completed in April 2021 using the same key terms as the indexed literature search. Due to the importance of national and historic contexts related to displacement that are specific to the USA, we excluded non-US-based articles as well as non-English language articles. Additionally, to make both searches feasible within time and resource constraints, we included in both only articles, resources, toolkits, or best practice guides that contained more than one strategy. Protecting against displacement in most place likely requires multiple strategies in concert with each other that employ a variety of policies. From the indexed literature review, study title and abstract screenings were completed by four screeners (AS, HD, KG, and KI), with at least two screeners reviewing each.

### Organization by Domains

We adapted an existing typology [36] to thematically characterize anti-displacement strategies. Domains from the existing typology included (1) preservation, (2) protection, (3) inclusion, (4) revenue generation, (5) incentives/disincentives, and (6) property acquisition. After reviewing all strategies, we added domains for (7) stabilization, (8) community engagement/education, and (9) cross-cutting strategies. Brief definitions of each domain are presented in Table 1.

**Table 1:** Typology of displacement prevention and mitigation strategies

Category	Definition	Examples
1. Preservation	Preserve existing affordable rental units	• Right to purchase laws • Demolition control
2.Protection	Help long-time residents who wish to stay in the neighborhood	• Employer assisted housing • Rent skewing
3.Inclusion	Ensure that a share of new development is affordable	• Inclusionary zoning policy • Density bonuses
4.Revenue generation	Harness growth to expand financial resources for affordable housing	• Tax Increment Financing (TIF) • Housing trust funds
5.Incentives/disincentives	Create incentives for developers of affordable housing, and/or discourage developers from increasing rents	• Anti-speculation taxes • Impact fees
6.Property acquisition	Facilitate acquiring sites for affordable housing	• Expropriation • Community land trusts
7.Stabilization	Stabilizing long time/historical residents by securing long-term housing	• Individual development accounts • Down payment assistance
8.Community engagement/education	Educate and engage with community members on factors related to affordable housing and displacement	• Coalition building • Awareness campaigns
9.Cross-cutting	Overarching thematic approaches related to displacement or affordable housing	• Health in all policies • Community planning

### Data Abstraction

We reviewed each included resource to identify any strategies that were proposed, implemented, or evaluated. We researched, as needed for presentation to public health audiences, strategies for which full descriptions were not included in the original sources. Three reviewers (KG, LR, and NS) abstracted data using a standardized form in Microsoft Excel and discussed and reconciled all discrepancies. Variables of interest included the strategy or measure identified, the definition of the strategy or measure, whether it related to residential and/or commercial displacement, and into which domain it best fit.

## Results

**Fig. 1:**
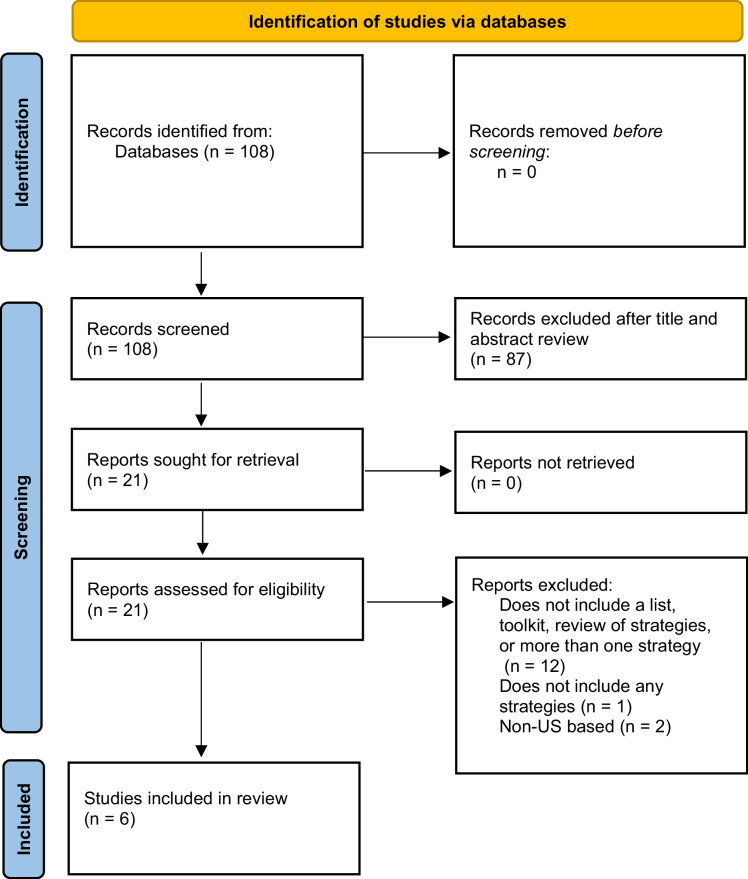
Preferred Reporting Items for Systematic Reviews and Meta-analyses (PRISMA) flow diagram to identify resources for displacement prevention and mitigation strategies from the indexed literature

Based on the search criteria, we identified 108 articles in the indexed literature and excluded 102 after abstract and full text reviews (Fig. 1). A total of six indexed articles met all eligibility requirements. The grey literature scan identified 280 potentially relevant documents, of which 224 were excluded because they did not include a list, toolkit, or review of strategies. The majority (70) of these 224 did not name specific anti-displacement strategies; 56 were news stories or blog posts; 51 dealt with measuring gentrification, displacement or risk; 47 were place-specific case studies, including three non-US places. We also excluded 38 documents that addressed single strategies. Thus, an additional 18 resources were identified from the grey literature scan for a total of 24 unique resources or articles. More information on each resource can be found in Table 2, including whether a resource was found in the peer-reviewed or grey literature, and the number of strategies per domain from each resource. The indexed literature search yielded only one journal publication and 5 academic products (i.e., dissertations, theses, or class papers), which we grouped with grey literature because they lacked peer review. Excluded grey and indexed literature (including two peer-reviewed papers) that only addressed single strategies would have added the following four strategies to Table 2: affordable housing residency preferences (indexed literature), qualified allocation plans (grey literature), eviction blockades (grey literature), and condo moratoria (grey literature). Though these were not added to stay consistent with our objectives and search strategy, this highlights the large percentage of strategies that were captured by our search. From these 24 resources, we identified and categorized 141 total anti-displacement strategies (Table 3).

**Table 2:** Selected resources for displacement prevention and mitigation strategies (*N* = 24 resources)

Resource	Type^a^	Preservation	Protection	Inclusion	Revenue generation	Incentives/disincentives	Property acquisition	Stabilization	Community engagement & education	Cross-cutting
		Number of Strategies								
1. “Greening in place: protecting communities from displacement” [37]	G	2	5	4	1	1	1	2	3	6
2. “All-In Cities Policy Toolkit” [38]	G	2	3	1	0	0	1	1	0	1
3. “Asian American and Pacific Islander Anti-Displacement Strategies” [39]	G	3	5	1	2	0	1	1	5	6
4. “Beyond Gentrification: Strategies for Guiding the Conversation and Redirecting the Outcomes of Community Transition” [40]	G	1	1	1	0	0	0	0	2	0
5. “Building American Cities Toolkit” [41]	G	3	7	2	1	0	4	4	2	0
6. “Community Control of Land and Housing” [42]	G	1	0	0	0	0	3	0	1	3
7. “Comparative Gentrification Policy” [43]	G	2	12	4	2	5	1	1	1	3
8. “Development Without Displacement” [44]	G	1	4	1	1	0	1	2	0	1
9. “Gentrification & Neighborhood Change” [45]	G	3	3	1	1	0	1	1	2	1
10. “Greening without Gentrification” [46]	G	2	7	3	1	2	1	4	2	1
11. “Healthy Development Without Displacement” [47]	G	6	8	5	2	3	2	4	5	5
12. “Housing Development Toolkit” [48]	G	0	2	4	0	4	0	0	0	0
13. “In the Face of Gentrification” [49]	G	2	2	1	0	1	3	2	0	0
14. “Keeping the Neighborhood Affordable” [50]	G	4	3	1	1	2	1	2	1	0
15. “Housing Policy Library” [51]	G	1	2	2	4	4	0	0	0	0
16. “Preserving, Protecting, and Expanding Affordable Housing” [52]	G	3	6	2	3	4	2	1	0	0
17. “Proven Local Strategies for Expanding the Supply of Affordable Homes and Addressing Cost Challenges” [53]	G	2	1	2	1	2	2	0	0	1
18. “Toward a socially acceptable gentrification: A Review of strategies and practices against displacement” [34]	P	4	8	4	0	3	5	4	3	2
19. “Urban Displacement Project” [54]	G	3	6	2	1	1	1	0	0	0
20. “What about Housing?” [55]	G	5	6	1	2	0	4	3	0	1
21. “Planning for Equitable Neighborhood Change” [56]	G	4	6	1	0	0	0	1	0	1
22. “Mitigating Displacement due to Gentrification” [57]	G	2	4	1	3	2	4	3	0	2
23. “Displacement Due to Gentrification” [58]	G	2	4	2	0	2	3	2	2	2
24. “Gentrification Response: A survey of strategies to maintain neighborhood economic diversity” [59]	G	3	5	1	1	2	1	0	0	0

^a^Whether the resource is peer-reviewed (P) or grey (G) literature.

**Table 3:** Strategies for the mitigation or prevention of displacement (*N* = 141 Strategies)

Domain	Strategy	Residential/commercial	Resource number(s)^a^
1. Preservation	1. Basement campaign: Effort to legalize basement units in places where they are illegal	Residential	3
	2. Code enforcement: Ensures landlords comply with health and safety codes so there is appropriate maintenance and repair in existing affordable housing stock	Both	1, 5, 7, 11, 13, 14, 20
	3. Demolition control: Prevents the demolition of a building	Both	11
	4. First right of refusal (aka first right of purchase/tenant opportunity to purchase) policies: If an owner decided to sell a building, current tenants or a local non-profit developer has the first right of refusal to purchase before a property can be sold to a private developer	Both	2, 4, 5, 8, 9, 11, 16, 18, 20–23
	5. Ground leases: Long term leases of city owned land that include an affordability requirement	Both	24
	6. Historic preservation: Cities can create preservation districts where houses, buildings, and other physical structures are preserved and regulated	Both	3
	7. Housing trust funds: An affordable housing production program to increase and preserve the supply of decent, safe, and sanitary housing by establishing dedicated streams of revenue to create or preserve affordable housing for low-income households. These can be used as gap financing in support of rehab or new development, and revenues are often tied to other market-driven programs	Residential	2, 7, 9–11, 13–21
	8. Limited equity cooperatives: Democratic, member-run cooperative organizations that limit the equity individual homeowners can accumulate, thus preserving long-term affordability	Residential	6, 9, 14, 18, 20, 22, 23
	9. Mechanisms to restrict use after sale: Deed restrictions and agreements that limit the use of formerly city owned land to affordable housing	Residential	24
	10. Mobile home rent control and park preservation: Places specific rent increase restrictions on the land rented by mobile home owners or the homes themselves	Residential	11, 19
	11. Preservation of federally subsidized affordable housing: Programs and strategies to encourage private owners of multifamily housing to provide affordable housing options to low-income households. Two types of subsidies include the Sect. 236 mortgage program and Sect. 8 subsidy program	Residential	14, 18, 21, 24
	12. Preservation of unsubsidized affordable housing: This type of housing is often affordable due to older age and limited amenities. Cities can use programs and initiatives to ensure this type of housing remains affordable	Residential	17
	13. Preserving farmlands & culture: Re-establish farmlands and economic opportunities for cultivation in places that historically grew crops, such as small family farms	Commercial	3
	14. Retain long-term affordable housing: Facilitate retention of projects subject to expiring use restrictions as permanent or long-term affordable housing	Residential	5, 20
	15. Single Room Occupancy (SRO) regulations: These house one or two people in individual rooms, with tenants typically sharing bathrooms and/or kitchens. These are often considered a form of permanent residence affordable for low-income individuals. SRO Preservation ordinances preserve or create new SRO units	Residential	10, 11, 19
	16. Small business creation and preservation (e.g., small business disruption funds): Resources and assistance can be provided to locally-owned small business owners to assist them in creating new jobs or keeping the ones they already have and increasing the earnings	Commercial	1, 10, 21
	17. Transfer of development rights: a zoning tool that can be used to help preserve existing affordable housing. Affordable housing sites can be “sending sites” and can sell the development rights to “receiving sites” where developers can build in ways not typically permitted by the zoning code (ex: build at a higher density or building height). Funds received by selling those rights can be used to upgrade units and preserve affordability	Residential	16
2. Protection	18. Anti-harassment laws/policies: Policies that prevent landlords from coercing tenants into leaving through negligence, intimidation, or buy-out offers	Both	5, 18, 24
	19. Commercial zoning regulations: Zoning regulations that protect small businesses, heritage, and cultural spaces	Commercial	8
	20. Condo conversion protections: These impose procedural restrictions (like notification requirements) and/or substantive restrictions on the ability to convert apartment units into condominiums (e.g. prohibiting conversions unless the city or regional vacancy rate is above a certain fixed amount)	Residential	1, 9–11, 16, 19, 21, 22
	21. Employer assisted housing: Employers provide local affordable housing to their employees living in the same community. This keeps workers close to where they live, which reduces transit costs, and may provide financial assistance to help homeowners build equity or help tenants meet rent payments	Residential	9, 15
	22. Fair housing: The Fair Housing Act protects people from discrimination when they are renting, buying, or securing financing for any housing. The prohibitions specifically cover discrimination because of race, color, national origin, religion, sex, disability, and the presence of children	Residential	11, 20
	23. First source hiring ordinance/Local Hiring Ordinances: First Source hiring ordinances ensure that city residents are given priority for new jobs created by municipal financing and development programs	Both	19
	24. Foreclosure prevention and assistance: Local programs that assist home owners (financially or otherwise) when they are at risk of foreclosure. These programs may be funded with federal grants	Residential	3, 5, 10, 11, 19, 20
	25. Housing choice vouches (Sect. 8 vouchers): The federal government’s major program for assisting very low-income families, the elderly, and the disabled to afford decent, safe, and sanitary housing in the private market. Participants are able to find their own housing, including single-family homes, townhouses and apartments	Residential	3, 7, 11, 14, 16
	26. Housing repair assistance programs: Provides loans to low-income homeowners to repair, improve or modernize their homes or grants to elderly low-income homeowners to remove health and safety hazards	Residential	5, 7, 13, 20–22
	27. Just cause (aka good cause) eviction policy: Protects tenants against unjust evictions	Both	1–3, 8, 10, 11, 16, 19–21
	28. Legal assistance to prevent eviction: Legal representation for those facing the possibility of eviction can help them understand their rights and stay in their homes	Residential	1, 2, 7, 18, 21, 24
	29. Reduced regulatory restrictions on second units/ADUs: Accessory dwelling units (ADUs) — also referred to as accessory apartments, second units, or granny flats — are additional living quarters on single-family lots that are independent of the primary dwelling unit. The adoption of ordinances, with reduced regulatory restrictions to encourage ADUs, can help increase a community’s housing supply. Since they cost less than a new single-family home on a separate lot, they are an affordable housing option for many low- and moderate-income residents. They also benefit homeowners by providing extra income that can assist in mitigating increases in the cost of living	Residential	3, 5, 7, 10–12
	30. Relocation assistance: Any family or individual that must move as a direct result of rehabilitation, demolition, or acquisition for a project in which Federal funds are used is eligible for relocation assistance under the Uniform Relocation Assistance and Real Property Acquisition Policies Act of 1970	Residential	1, 5, 18, 24
	31. Renovation assistance: Complementary to home purchase assistance in the case of Project Renewal	Residential	18
	32. Rent control/stabilization policies: Government control and regulation of the amounts charged for rented housing, typically determined by a formula or a body of experts on the issue	Residential	1, 5, 7, 8, 10, 11, 13, 14, 16, 18–24
	33. Rent review board and/or mediation: Rent review boards mediate between tenants and landlords on issues related to rent increases and encourage them to come into voluntary agreement. As mediators, the board normally does not make a binding decision in the case	Residential	10, 19
	34. Rent skewing: A system through which higher-income residents subsidize the rent of lower-income ones	Residential	18
	35. Rent subsidies: Use public funds as a tenant protection strategy	Residential	18, 23
	36. Right-to-stay/return: With a building renovation or redevelopment, developers must offer existing tenants new apartments at comparable rates in the new building	Residential	7, 8
	37. Source of income protection: Local ordinances that prohibit owners from refusing to rent to an applicant based on their source of income	Residential	24
	38. Tax abatement policies/property tax caps: Tax abatement policies fix property taxes as land values begin to rise. Residents and business owners who meet specified criteria (e.g., income, length of time in the neighborhood) can apply for fixed property taxes. The freeze can remain in place as long as the homeowner continues to qualify	Residential	4, 5, 7, 9, 10, 12, 14–16, 21–23
	39. Tax exemptions: Taxpayers may be granted an exemption from a certain tax in order to maintain property affordability	Residential	5, 11, 16, 18
	40. Transit fee elimination for school-aged children: school-aged children are given access to free public transit during typical commute times. This can help students access their school even if they must move to different places in the city	Residential	7
	41. Transit subsidies for low-income households: Policies that provide transit subsidies for lower-income households or allow children to bus to school for free	Residential	7
	42. Transit-oriented planning and development: Creating mixed-use, walkable communities around new or existing public transit stations can lower residents’ transportation costs and connect them with jobs or services	Both	2, 3, 7, 17
	43. Transportation affordability calculators: State Qualified Allocation Plans could prompt developers of Low Income Housing Tax Credit properties to set rents at a particular percentage of area median income minus half the cost of monthly commutes from that location to downtown. Local subsidy programs and inclusionary zoning can follow a similar procedure when setting rents	Both	7
	44. Two year right to renew lease standard: Requires a lease to have minimum two-year right-to-renew without a rent increase. Alternatively, they can encourage landlords to adopt model 2-year right-to-renew leases written by the municipality, in exchange for reduced inspection fees or other incentives	Residential	7
	45. Unified tenant screening report: Common application and criteria for rental units negotiated by landlords and tenants’ groups	Residential	20
3. Inclusion	46. Affordable housing provision: Language included in ordinances, contracts, or other legal documents that require the inclusion of affordable housing	Residential	18
	47. Density bonuses: Allows developers of market-rate housing to build higher-density housing in exchange for having a certain portion of their units offered at affordable prices	Residential	10–12, 15–17, 19,
	48. Equitable green development overlay zones: Superimposes new zoning codes to an existing zoning map. Cities can include a package of strategies to create equitable green development	Both	1
	49. Inclusionary zoning policy: An affordable housing tool that links the production of affordable housing to the production of market-rate housing	Residential	1–5, 7–24
	50. Land value recapture: When government actions like rezoning or land use changes increase profitability for developers, cities can require than anyone building in that area to include a certain number of affordable units (inclusionary housing)	Residential	1, 10–12, 18
	51. Make room for local business in new developments: Require developers set aside space for independent local businesses in new developments	Commercial	7
	52. One-for-one (one-to-one) replacement: All occupied and vacant occupiable lower-income dwelling units that are demolished or converted to a use other than as lower-income dwelling units in connection with an assisted activity must be replaced with comparable lower-income dwelling units	Residential	1, 5, 7, 11, 18, 23
	53. Regional affordable housing coordination: A regional policy that would require all states, cities, or towns within a region to commit to meeting housing goals. The affordable housing goals must balance what is feasible with what is necessary to meet demand at all income levels, with negative consequences if a community failed to participate in the communal goal-setting process or failed to meet its goal	Residential	7
	54. Remove density restrictions, allow multifamily zoning: Zoning or density restrictions can make it difficult to increase the supply of affordable housing. By changing the zoning code and allowing higher density, more affordable units can be built	Residential	11, 12
4. Revenue generation	55. Commercial linkage fee: Fees that are charged to developers of commercial spaces like offices or retail spaces for the creation of affordable housing	Commercial	8, 10, 16, 17, 19, 20, 22, 24
	56. Community development loans: Provides access to capital targeting non-profit groups. Loans support equitable community development projects, such as affordable housing	Commercial	3
	57. Dedicated revenue sources: Ongoing source of funding that can come from specific taxes and fees dedicated to affordable housing that is often kept in a housing trust fund	Residential	15
	58. General obligation bonds for affordable housing: The proceeds from these government-issued bonds can be used to fund affordable housing programs	Residential	11, 15, 16
	59. Housing levies: A property tax assessment which generates money designated toward affordable housing projects	Residential	9, 22
	60. Increase use of multifamily private activity bonds to draw down 4 percent LIHTCs: States can issue a certain amount of tax-exempt private activity bonds each year which can go to activities with public benefit such as affordable housing. When used for affordable housing, the developments automatically receive federal 4 percent Low-Income Housing Tax Credits	Residential	15
	61. Industry dividends to help fund programs: A city purchases shares in a company headquartered in the city at a discounted rate. Annual earnings are put into a dedicated fund to combat displacement	Residential	7
	62. Reinvest criminal justice spending in affordable housing: Cities could reinvest a portion of the general funding granted to criminal justice and public safety in affordable housing construction programs, especially supportive housing	Residential	7
	63. Small business & stabilization microloans: Financial products geared towards small businesses can help them obtain the capital they need which is often unavailable from mainstream banks and financial institutions	Commercial	3, 5
	64. Tax Increment Financing (TIF): Revenue generated by borrowing against the projected increased property tax collection within designated redevelopment (urban renewal) districts. All or a portion of the tax increment can be set aside for affordable housing preservation and production	Residential	1, 14–16, 20, 22
	65. Toll and parking fees: Invest revenue generated from toll lanes and parking fees in active transportation infrastructure, affordable housing trust funds, park development, etc	Residential	11
5. Incentives/disincentives	66. Anti-speculation taxes: Taxes to discourage speculative investors, or “house flippers,” from buying and rapidly reselling properties	Residential	18
	67. Competitive funding for parks requiring or incentivizing anti-displacement strategies: Funding agencies can require or incentivize applicants to include anti-displacement strategies in their applications	Both	10
	68. Establish by-right development: Allows projects that meet specific requirements to be administratively approved, streamlining the review process and allowing for more efficient development	Residential	7, 12
	69. Excise taxes on homes sold to higher-income buyers: Taxes that reduces the incentive to "upgrade" housing by charging an excise tax on home purchase transactions in gentrifying neighborhoods	Residential	7
	70. Expedited development review and permitting: Fast tracks the development review and permitting process for projects that meet defined displacement prevention and health equity criteria	Residential	12, 15, 16
	71. Low-income housing tax credits (LIHC): Federal program that provide funding for the development costs of low-income housing by allowing an investor (usually the partners of a partnership that owns the housing) to take a federal tax credit equal to a percentage of the cost incurred for development of the low-income units in a rental housing project	Residential	14, 23
	72. Luxury housing taxes: property tax on luxury homes. The taxes could discourage the development of new luxury housing and/or could generate funds to assist with affordable housing	Residential	18
	73. Permit streamlining/fee reduction: Reduce pending permit applications, reduce fee for permitting	Both	11
	74. Property transfer taxes: A tax that is imposed on a change in ownership of real estate where the title is transferred from seller to buyer. It is equal to a percentage of the sale price	Both	18, 22, 23
	75. Reduced or eliminated parking requirements: Reduces the parking minimum for affordable housing projects, increases requirements for bicycle parking, allows underused private parking lots to be open to the public, and separates parking fees from rental leases. Reduce off-street parking minimums	Both	11, 12, 15–17
	76. Residential linkage fees/impact fees: One-time charges designed to cover the costs of building infrastructure to support new development, such as water lines, sewer lines, and schools. By reducing or waiving these fees for newly developed affordable housing, localities can provide incentives for developers to provide affordable housing	Residential	1, 7, 10, 11, 15–17, 19, 24
	77. Split-rate taxes: Tax structure that separates property taxes into a higher rate for land and a lower rate for buildings. This encourages improvements to buildings and discourages land speculation	Both	14
	78. State tax credits or subsidies for affordable housing: States can provide a credit against state income taxes to reduce project financing costs for affordable housing	Residential	15, 16, 22, 24
	79. Tax on rent increase: The amount of property tax paid by a landlord would be tied to the percent that the rent on a property increases from one year to the next. If the rent increased less than the average percent increase for the entire metropolitan area, then the landlord would pay no additional tax	Residential	7
	80. Tax vacant land or donate it to nonprofit developers: Localities increase the fees the longer a property remains vacant, which encourages lot owners to put their properties to more productive use, such as redevelopment. Once vacant property has been identified, jurisdictions are able to take action to combat the lost revenue and blight that come with vacant property by taxing vacant land or donating to non-profit developers	Residential	7, 12, 13
6. Property acquisition	81. Acquisition and rehabilitation of privately owned vacant and underutilized property: Transforming vacant and underutilized privately owned property into affordable housing can increase the supply of affordable homes. Jurisdictions can offer developers low-cost financing to incentivize them to acquire privately owned vacant sites or buildings and turn them into affordable housing developments	Residential	5, 11, 13, 17, 20
	82. Community land trusts: Nonprofit, community-based organizations designed to ensure community stewardship of land	Both	1, 2, 6–10, 13, 14, 18–20, 22, 23, 24
	83. Expropriation: Taking land from private market and putting it in public hands	Residential	18
	84. Infill development: The development of vacant or under-used parcels in areas that were previously built	Both	5, 13, 18, 22, 23
	85. Land banking/using publicly owned land: Nonprofit or public entities with legal authority to acquire and remediate blighted properties; later act as a broker to repurpose land for best use (e.g., affordable housing, parks)	Both	5, 6, 16–18, 20, 22, 23
	86. Property acquisition funds: Funds to facilitate the purchase and holding of properties for affordable housing development. One approach is a revolving loan fund that provides low-interest-rate loans to nonprofit organizations so they can acquire property for development or redevelopment of affordable housing. Through acquisition funds, affordable developers can access low-interest capital more quickly than through other public sector funding sources	Residential	5, 16
	87. Public land disposition: Localities can make publicly owned land available at reduced or no cost to facilitate affordable housing. Selling public land at a discount can be a strong incentive for mixed-income housing in hot housing markets, and it can provide major cost savings for all affordable housing developments in communities where land prices are high	Residential	11, 20, 22
	88. Public land use for family housing: Prioritize public land (e.g., parking lots) for affordable housing and mixed-use	Residential	3
	89. Resident owned communities: Democratic, member-run cooperative organizations that own the land in manufactured housing communities, thus protecting against displacement, poor conditions, and exploitative management practices	Residential	6, 18
7. Stabilization	90. Forgivable loans for home improvements: a type of financial assistance that is designed to help homeowners make improvements to their homes. Once the project is complete and meets certain requirements, the loan is forgiven	Residential	10
	91. Asset building programs: Help people from low and middle income communities build assets to help stabilize families, increase agency, and strengthen communities	Residential	11
	92. Home ownership purchase assistance: Provides affordable mortgage financing to eligible home-buyers	Residential	5, 10, 11, 13, 18, 20, 22, 23
	93. Homelessness diversion: Wrap-around services to help vulnerable and low-income people find and keep housing	Residential	3
	94. Housing first: Approach that prioritizing providing permanent housing to people experiencing homelessness	Residential	20
	95. Housing rehab and preservation: The preservation process can allow faster, easier, and cheaper maintenance of existing properties than building new. It can also help low-income communities with maintenance of units, including weatherization and improved accessibility	Residential	1, 5, 9, 11, 13
	96. Individual development accounts: Individual development accounts (IDAs) are matched savings accounts designed to help low-income and low-wealth families accumulate savings for long-term	Both	14, 22
	97. Lease-to-own programs: Tenants have the opportunity to purchase their rental property by turning lease payments into equity	Both	5, 8, 23
	98. Living wage policies: Living wage standards are for city employees, government contractors, and companies receiving public subsidies, to ensure that public spending creates good family-supporting jobs	Both	1
	99. Local job creation: the creation of jobs for local residents in a city or town	Both	5, 10, 18
	100. Location efficient mortgages: Offer mortgages for properties located in central, walkable areas	Both	14, 18, 22
	101. Minimum wage laws: Local minimum-wage policies can lift families out of poverty and promote economic security	Both	2
	102. Minority and low-income contracting: Policies and programs to increase opportunities for minority-owned and other emerging small businesses. Most of these programs focus on the construction industry, because it receives a large amount of public money and offers well-paying jobs for people without advanced education	Both	7, 21
	103. Progressive real property taxes: Property taxes that are based on the taxpayer’s income. Lower income earners would pay at a lower tax rate compared to higher income earners	Residential	18
	104. Rental assistance demonstration: Offers a solution that converts public housing subsidies into a form that can be used as the basis for securing private financing and can be combined more easily with other subsidies. The most likely beneficiaries of the RAD program are people currently living in public housing (or in some cases, other forms of HUD-assisted housing) or very low-income households that may apply for vacant units	Residential	11, 16
	105. Risk mitigation fund for displaced renters: Dedicated fund of money to assist renters who are being displaced or at risk of being displaced. Cities and organizations can set up these funds to support renters in a variety of ways (ex: relocation assistance, emergency assistance for a crisis)	Residential	10
	106. Down payment assistance: Programs that help households attain homeownership through financial support for closing costs and various-sized down payments	Residential	20
	107. Home ownership protection policies: Financial assistance policies that can help support low-income homeowners and help them stay in their homes	Residential	8
8. Community engagement and education	108. Awareness campaigns: Community organizations can increase support for affordable housing policies through awareness campaigns that build on the community’s shared narrative in order to mobilize people to action	Both	4
	109. Coalition building: A way to share information and to collaborate with others to advance a common cause	Both	9
	110. Community education and empowerment: A collection of strategies that can: (1) Provide education on governance, land use planning, and policymaking processes; (2) Develop media/communications campaigns reinforcing that community conditions shape health; (3) Educate and organize youth to influence land use planning and policymaking in their own communities; (4) Equip local residents, youth, and others with strong community ties to become civic leaders and decision-makers	Both	1, 3, 5, 11
	111. Community engagement: The process of working collaboratively with groups of people who are affiliated by geographic proximity, special interests, or similar situations with respect to issues affecting their wellbeing	Both	11
	112. Community organizing and representation in the planning process: Ensures that residents have easy access to information and gives low-income communities representation on planning committees, transportation boards, and boards of architectural review	Both	3, 7, 11, 23
	113. Dealing with NIMBYism: NIMBY stands for Not in My Backyard and includes people who object to having something they perceive as undesirable in their neighborhood. NIMBY attitudes can make it difficult to build new affordable housing in a community. Education and relationship building before a proposed project begins can help	Both	9
	114. Educating providers: (1) Increase knowledge about displacement and health equity concepts and strategies among community-based organizations, government agencies, and policymakers; (2) provide continuing education/certification maintenance opportunities that address displacement and health equity concepts	Both	11
	115. Home ownership education and counseling: Programs that educate prospective buyers to improve their odds of buying and staying in a home	Residential	4, 5, 10, 14, 23
	116. Human overlay assessment: Assesses the human impacts of neighborhood development through surveys and focus groups. Utilizes participatory mapping and photography to bring in local voices into the planning decision-making process	Both	3
	117. Language access: Provides culturally sensitive, multilingual communication strategies so non-English speaking residents can understand, participate in, and engage with projects in their community	Both	1
	118. Participation: Resident involvement in anti-displacement efforts	Both	6, 18
	119. Registered community organizations: Community-based organizations that organize and serve on behalf of community members. Can create a process to ensure community member voices are heard	Both	3
	120. Social movements: Collective action in pursuit of a specific political or social issue	Both	18
	121. Stakeholder notification: System that alerts community members to new development activity in their neighborhood	Both	1
	122. Tenant counseling and educating the local population: Tenant counselors can help tenants better understand their rights in a potential displacement situation	Residential	3, 10, 11, 18
9. Cross-cutting	123. Accountability and monitoring: Determines if a project is meeting goals through set metrics and evaluation	Both	1
	124. Area-wide no net loss assessment: Determines the change in the number of affordable units over a period of time. If there is a loss, communities can use that information to implement appropriate strategies to increase affordable housing	Residential	1, 21, 22
	125. Arts-driven placemaking: Creative arts and placemaking can preserve and create cultural assets. It also can draw people to neighborhoods, provide spaces for economic growth and sustainability	Both	3
	126. Ballot measures: Also known as a “proposition,” jurisdictions can introduce affordable housing measure on a ballot which is voted on by residents during an election	Residential	17
	127. City-wide anti-displacement plan: Specific plan that can be integrated into a Comprehensive Plan that is designed to prevent displacement and increase access to affordable housing	Both	3, 23
	128. Collaboration of service providers for high needs populations: City officials coordinate regular meetings of service providers for high needs populations such as lower income, elderly, immigrant, and homeless populations, to facilitate regional coordination	Both	7
	129. Community benefits agreement: Deals between developers and coalitions of community organizations, addressing a broad range of community needs. These agreements are safeguards to ensure that affected residents share in the benefits of major developments	Residential	1, 3, 6–11, 18, 20, 22, 23
	130. Community planning: Include specific anti-displacement measures in comprehensive or general plans and community plans	Both	1, 3
	131. Cultural eco-district: A plan for a neighborhood that ensures its economic, environmental, and cultural livelihood	Both	3
	132. Develop data to support community control of land and housing and combat displacement: Improving the capacity to track, report, interpret, and analyze data related to affordable housing and related strategies	Both	6
	133. Displacement impact report: Analyzes and describes potential displacement impacts associated with community development. Can help with understanding needs for mitigation strategies	Both	1
	134. Healthy Neighborhood Strategy/Health in All Policies (HIAP) ordinance/resolution: A health in all policies ordinance/resolution integrates health considerations and performance standards into all government practices	Both	2, 3, 11
	135. Joint development of parks, open space, and housing: Occurs when parks and recreation partners join with affordable housing partners to create green development alongside affordable housing	Residential	1
	136. Multi-sectoral coalitions: Establish and fund multi-sector coalitions to take collective action in support of healthy development without displacement	Both	11
	137. Municipal land use control: The regulation of public and private land through government ordinances, codes, and permits (ex: zoning, building codes, housing codes, etc.)	Both	18
	138. Place racial equity at the center of strategies for community control of land and housing: Organizations and individuals who work on affordable housing can use a racial equity lens and keep in mind the current consequences of historic racist policies	Both	6
	139. Project scoring and selection processes: Build health equity and displacement prevention criteria into project scoring and selection processes	Both	11
	140. Rental registry for quality monitoring: Database where landlords register their properties. Often linked with code enforcement and building inspections	Residential	7
	141. Transportation investment: Invest in transportation infrastructure such as buses and trains	Both	11

^a^Resource number(s) reflect the items listed in Table 2

Six of the nine domains were conceptually tied to affordable housing, and a similar majority of the strategies (62%) were categorized within those six; only stabilization, community engagement/education, and cross-cutting strategies addressed other factors. Of the 141 strategies, 81 addressed residential displacement, 53 both residential and commercial displacement, and seven commercial displacement only. More detail on each domain is provided below.

### Domains of Anti-displacement Strategies

#### Preservation (*n* = 17)

These strategies aim to preserve existing affordable rental units *despite* increasing property values [36]. This could come in two forms, either rent-restricted affordable units or unsubsidized affordable units. Conceptually, these strategies protect against displacement by ensuring a set amount of affordable housing options, which may or may not be intended for long-term residents. An example strategy would be preservation of federally subsidized affordable housing which include programs designated to preserve affordable housing units. Most strategies in this domain addressed residential displacement (*n* = 10), five addressed both residential and commercial displacement, and two addressed only commercial displacement.

#### Protection (*n* = 28)

These strategies focus on helping long-time renters, typically low-income households, who wish to stay in the neighborhood [36]. These strategies include legal protections for long-time renters, along with voluntary practices. An example is anti-harassment laws, which prevent landlords from coercing tenants into leaving. Protection strategies may also mitigate the impacts of displacement by helping long-time residents stay connected to neighborhoods. An example of protection through mitigation practices is transit subsidies for low-income households, which reduce barriers of long-time and low-income residents to stay connected to a neighborhood, despite the possibility of being physically displaced. Most of these strategies focus on residential displacement (*n* = 22); three targeted both residential and commercial displacement, and three only commercial displacement.

#### Inclusion (*n* = 9)

These strategies focus on ensuring that new development or a share of new development will be affordable for the long term (30 or more years) [36]. This strategy is broad and addresses displacement by creating affordable housing possibilities in the context of new development. New affordable housing creates more options for long-time residents to stay in their neighborhoods. Inclusionary zoning policies, which create expectations of developers to include affordable housing, are the best known of these strategies and almost synonymous with the definition of this domain. Though most inclusionary policies are mandated, there are examples of voluntary inclusionary practices by developers. Most policies in this domain targeted residential displacement specifically (*n* = 7); one targeted both residential and commercial displacement, and one commercial displacement alone.

#### Revenue generation (*n* = 11)

This domain is also broad and leverages the growth and financial resources connected to new development and redevelopment towards ensuring funding for affordable housing [36]. Like inclusion, revenue generation aims to ensure a share of affordable housing possibilities within new development. Conceptually, this strategy creates more potential options for long-time residents to stay in their neighborhoods despite rising prices. A primary example of this is Tax Increment Financing (TIF), which uses the expected increases in property tax due to new development and public improvements to fund the new development itself. TIF mechanisms that establish requirements to use a portion of the funding for affordable housing can be a displacement prevention strategy. Most of the strategies identified in this domain targeted residential displacement (*n* = 8); three targeted commercial displacement.

#### Incentives/disincentives (*n* = 15)

This domain includes strategies that encourage communities and developers to create affordable housing by providing incentives that are often financial [36]. We added disincentives to the original typology to encompass strategies that discourage communities and developers from rapidly increasing rents or engaging in speculative housing strategies. Both strategies act against displacement by creating or maintaining affordable housing options. For example, state tax credits for affordable housing can reduce project financing costs and thus provide an incentive to develop affordable housing. Inversely, a tax on rent increases would be a disincentive to landlords from large rent increases. Ten of these strategies focused solely on residential displacement, and the remainder on both residential and commercial displacement.

#### Property acquisition (*n* = 9)

These strategies facilitate acquiring funding or sites for affordable housing, usually by nonprofit or community organizations [36]. Property acquisition strategies help accumulate funding or give priority to the public or nonprofit sector to acquire property that will be dedicated to affordable housing. These help protect against displacement by ensuring that housing will either stay or become affordable. An example of this is prioritizing public land for affordable multi-family housing and mixed use development. Two-thirds of the policies identified in this domain focused on residential displacement (*n* = 6), the remainder commercial displacement.

#### Stabilization (*n* = 18)

This domain includes strategies to increase stability for existing residents by securing and providing resources to become a home owner and help to maintain home ownership. These strategies may assist residents in pursuing home ownership, and empower residents with resources and assistance to stay in their neighborhoods. Stabilization efforts work towards ensuring long-term stability in housing, thus helping to protect against both the physical and cultural displacement of homeowners. One example is home ownership protection policies, which are financial assistance policies that help low-income homeowners to keep their homes. All stabilization strategies we identified targeted residential displacement and seven also targeted commercial displacement.

#### Community engagement/education (*n* = 15)

These strategies focus on educating and engaging with community members. Several relate to educating community members on affordable housing and homeownership processes. Others aim to ensure that long-time residents have a voice in any development or planning process. Examples of the latter include community organizing and supporting resident representation in planning processes. A majority of the strategies in this domain targeted both residential and commercial displacement; only two were focused solely on residential displacement.

#### Cross-cutting (*n* = 19)

This domain includes overarching policies or themes related to displacement or affordable housing. As such, most strategies targeted both commercial and residential displacement with only five focused solely on residential displacement. Strategies in this domain primarily targeted systemic issues related to displacement and frequently health disparities. One example is the practice of Health in All Policies (HIAPs), which strives to integrate health considerations of all communities into government practices.

## Discussion

This review was conducted in response to inquiries from community-based public health practitioners, based on concerns from the populations they serve, who are often disproportionately affected by disparities in physical activity. It provides a comprehensive list of anti-displacement strategies and well-categorized resources for learning more about how to address displacement concerns in the context of built environment changes to support healthy lifestyles. To characterize a wide range of anti-displacement strategies, we adapted an analytical framework [36] and added domains to encompass the wide range of strategies we identified. We then placed each strategy within one of nine domains and characterized each domain with a brief description, including relevance for residential and commercial displacement domains.

This scoping review comprehensively compiles existing strategies and resources with potential to protect against displacement, beyond affordable housing. Other reviews have focused solely on affordable housing [33], which is related to displacement but does not fully account for its complexities. Though not within the scope of our review, it is important to note the impact of displacement due to natural disasters, conflict, and crises. The current review provides high-level descriptions for a large number of strategies with potential to protect against displacement and makes these more easily accessible for a wide range of audiences including community-based public health practitioners.

It is important to note the limitations of the present study, including the potential exclusion of strategies based on our review’s objectives. Additionally, the available evidence limited our ability to further characterize the identified strategies including being able to present information on facilitators and barriers of these anti-displacement strategies, as well as the main actors involved, among other details. This may be partially due to the lack of indexed peer-reviewed manuscripts available. However, our project had multiple strengths, comprising a thorough review (including indexed and grey literature) and coding by multiple researchers. The greatest strength is the responsiveness to a need expressed regularly by public health practitioners to address community concerns about gentrification and displacement in connection with built environment changes. Our review provides a single, well-categorized inventory that can help practitioners appreciate the wide range of potential strategies. When built environment approaches are being considered, public health practitioners can then engage local expertise and lived experiences to select policies and practices best suited to increase health and equity in their local context.

We identified several remaining gaps upon completion of this study. One immediate need is further exploration of the relationship between gentrification, displacement, and projects aimed at improving the built environment for increased physical activity and healthy lifestyles. A majority of articles and resources found by our review came from the grey literature, highlighting a lack of peer-reviewed research on anti-displacement strategies. We did not conduct an evidence review, but we identified a prior systematic review of displacement prevention or mitigation studies published between 1980 and 2016 by Ghaffari et al. This review referenced 52 studies, of which we examined the full texts for 49; the remaining three studies were inaccessible by our library (2) or published in a language other than English (1). Of the 49 studies we examined, only 8 reported results from any evaluation and only 2 used quantitative, hypothesis-testing methods. Thus, our examination of a prior review with a different focus than ours highlights a gap related to public health evidence on the impact of anti-displacement strategies. Further evaluation of impacts on equity could also be useful to practitioners and community members faced with choosing among a wide variety of strategies.

Some strategies may be better suited than others when accounting for variations in for communities’ land use regulations, and geographic, social, political, or population characteristics. Gentrification itself has been highlighted as a socially and economically beneficial change to an area that promotes inclusion and social mixing [60]. However, gentrification may lead to detrimental effects on a community including the potential to undermine social cohesion [61]. Some strategies may even exacerbate displacement within certain contexts. For example, historic preservation was identified in our review as an anti-displacement strategy, but has raised concerns about encouraging gentrification and displacement in some contexts [62–65]. Gaps still exist in which anti-displacement strategies might work best and promote equity in which locations.

Given widespread community concerns and some emerging evidence, preventing displacement is an important priority for health equity. As a result, it makes sense to present known practices for mitigating and preventing displacement while acknowledging their possible limitations [66]. It is also important to continue research to understand the dynamics of gentrification and displacement along with the impact of mitigation and prevention strategies.

More expansive and nuanced theoretical frameworks could inform research on the processes and pathways through which built-environment approaches influence health, health equity, gentrification, and displacement. Some theoretical work has overlooked differential impacts of built environment initiatives by income, race, or other social dimensions [17]. New theoretical frameworks could clarify knowledge gaps and promote exploration of disparate impacts for communities with people of lower incomes or from racial and ethnic minority groups [67–69]. Guided by more inclusive theories, future research could include harms that are distinct from the loss of a home. Frameworks are needed that include emotional attachment to places and social processes that attach intrinsic value to a specific geographic space [70, 71]. Furthermore, accounting for and centering the lived experience of people and communities experiencing gentrification or displacement is essential to integrate into these new models. Given the time horizon of the health effects associated with built environment and economic changes, frameworks that consider the life courses of places and people may also be helpful [72].

Though gentrification and displacement of communities should not be overlooked, it is also important to note the usefulness of land use, community planning, and improved transportation systems for promoting health equity and combating racial inequities. There is recent expert consensus that transportation and land use policies that improve neighborhood accessibility, including complete streets policies are vital for health equity, when implemented with policies that protect communities including zoning reform and anti-displacement strategies [73–75]. Zoning reform has the potential for significant impact on health equity as it can influence community design that is beneficial for health, and simultaneously include protections for long-term residents (e.g., inclusionary zoning, affordable housing).

## Conclusion

In this work, we have assembled and organized an extensive menu of strategies to prevent or mitigate displacement in communities considering or undergoing changes that include built environment interventions intended to promote physical activity and health. Built environment interventions hold substantial promise to improve health and reduce health disparities, but only if people of lower income and/or from racial and ethnic minority groups, are able to remain and benefit from new development or infrastructure investments. The ability to design, create, and maintain amenities that encourage active living while preserving affordable, stable communities depends on creatively combining local knowledge with context-sensitive anti-displacement strategies. Ultimately, policy solutions to health disparities will require equity to be a central goal.
